# Editorial: Recent Advances in Recombinant Antibody Therapeutics and Diagnostics for Infectious Diseases

**DOI:** 10.3389/fpubh.2022.876889

**Published:** 2022-03-14

**Authors:** Theam Soon Lim, Michael Hust, Livia Visai

**Affiliations:** ^1^Institute for Research in Molecular Medicine, Universiti Sains Malaysia, George Town, Malaysia; ^2^Technische Universitat Braunschweig, Institut für Biochemie, Biotechnologie und Bioinformatik, Berlin, Germany; ^3^Department of Molecular Medicine, Centre for Health Technologies (CHT), Consorzio Interuniversitario Nazionale per la Scienza e Tecnologia dei Materiali, Unità Operativa di Pavia, University of Pavia, Pavia, Italy; ^4^Medicina Clinica-Specialistica, Unità Operativa di Ricerca 5 Laboratorio di Nanotecnologie, Istituti Clinici Scientifici Maugeri, Istituto di Ricovero e Cura a Carattere Scientifico, Pavia, Italy

**Keywords:** phage display, antibody, infectious disease, therapeutics, diagnostics

The recent pandemic has shown the threat posed by infectious diseases to the general population. Throughout the pandemic, the importance of vaccines, therapeutics, and diagnostics was evident to aid in the management of the COVID-19 outbreak. This is true for any infectious disease where diagnostics is critical to inform a person of their infection status and therapy to aid in the recovery. The use of antibodies in the development of diagnostics and therapeutics was evident with the surge of antibody development projects during the recent pandemic. This Research Topic focuses on the recent development of recombinant antibody technology for infectious diseases.

The role of recombinant antibodies in infectious diseases was clearly described by Roth et al., where they detailed the application of phage display for the development of antibodies against various infectious agents. The review provided a comprehensive view on the technology and its potential applications. Additionally, Spencer et al. discussed the role antibodies play in the HIV prevention. They were eloquent in describing the challenges faced for moving broadly neutralizing antibodies from discovery to clinic. This has always been seen as a major hurdle for any antibody project. The breadth of coverage conferred by antibodies was highlighted with the review by Longoni et al. where they discussed the role antibodies play in protozoan infections.

Moreira et al. provided an account of how phage display is applied to identify monoclonal antibodies for the detection of *Listeria monocytogenes*. Liu et al. exhibited the potential of phage display to be used for the development of antibodies against chikungunya virus. The work pays homage to the variability of different recombinant antibody formats that can be engineered. The work focused on the stability of domain antibodies against chikungunya. The application of recombinant DNA technology to engineer improved characteristics can also be witness by the publication from Schneider et al. They showed how lyophilized Fc-fused scFv formats can aid in prolonging the shelf life of the antibodies which is very essential for the applications of antibody therapeutics, but also diagnostics, in countries where a constant cold chain for transport and storage cannot be guaranteed.

With the lessons learned from the latest pandemic, it is evident that the role of recombinant antibodies is critical in times of health crisis for the rapid development of diagnostics and therapeutics. Therefore, constant advancements in technology not only for the isolation and identification of monoclonal antibodies against infectious diseases is crucial, but also for the further development of diagnostic approaches as well as preclinical development.

## Author Contributions

All authors listed have made a substantial, direct, and intellectual contribution to the work and approved it for publication.

## Conflict of Interest

The authors declare that the research was conducted in the absence of any commercial or financial relationships that could be construed as a potential conflict of interest.

## Publisher's Note

All claims expressed in this article are solely those of the authors and do not necessarily represent those of their affiliated organizations, or those of the publisher, the editors and the reviewers. Any product that may be evaluated in this article, or claim that may be made by its manufacturer, is not guaranteed or endorsed by the publisher.

